# A visceral organ function-focused therapeutic strategy using a 6-hour time window for patients with acute type a aortic dissection complicated by mesenteric malperfusion

**DOI:** 10.1186/s13019-024-02634-w

**Published:** 2024-04-05

**Authors:** Ling-chen Huang, Shuang-kun Chen, Hua Peng, Xi-jie Wu

**Affiliations:** 1 Department of Surgery, Fuwai Hospital, National Center for Cardiovascular Diseases, Chinese Academy of Medical Sciences and Peking Union Medical College, 167# Beilishi Road, Beijing, 100037 China; 2 Department of Cardiac Surgery, School of Medicine, Xiamen Cardiovascular Hospital of Xiamen University, Xiamen University, 2999 Jinshan Road, Huli 25 District, Xiamen, 361008 China

**Keywords:** Acute type a aortic dissection, Mesenteric malperfusion, Treatment strategy, 6-hour time window, Visceral organ function

## Abstract

**Background:**

Acute type A aortic dissection (ATAAD) complicated by mesenteric malperfusion is a critical and complicated condition. The optimal treatment strategy remains controversial, debate exists as to whether aortic dissection or mesenteric malperfusion should be addressed first, and the exact time window for mesenteric ischemia intervention is still unclear. To solve this problem, we developed a new concept based on the pathophysiological mechanism of mesenteric ischemia, using a 6-hour time window to divide newly admitted patients by the time from onset to admission, applying different treatment protocols to improve the clinical outcomes of patients with ATAAD complicated by mesenteric malperfusion.

**Methods:**

This was a retrospective study that covered a five-year period. From July 2018 to December 2020(phase I), all patients underwent emergency open surgery. From January 2021 to June 2023(phase II), patients with an onset within 6 h all underwent open surgical repair, followed by immediately postoperative examination if the malperfusion is suspected, while the restoration of mesenteric perfusion and visceral organ function was performed first, followed by open repair, in patients with an onset beyond 6 h.

**Results:**

There were no significant differences in baseline and surgical data. In phase I, eleven patients with mesenteric malperfusion underwent open surgery, while in phase II, our novel strategy was applied, with sixteen patients with an onset greater than 6 h and eleven patients with an onset less than 6 h. During the waiting period, none died of aortic rupture, but four patients died of organ failure, twelve patients had organ function improvement and underwent surgery successfully survived. The overall mortality rate decreased with the use of this novel strategy (54.55% vs. 18.52%, *p* = 0.047). Furthermore, the surgical mortality rate between the two periods showed even stronger statistical significance (54.55% vs. 4.35%, *p* = 0.022). Moreover, the proportions of patients with sepsis and multiorgan failure also showed differences.

**Conclusions:**

Our novel strategy for patients with ATAAD complicated by mesenteric malperfusion not only improves the surgical success rate but also reduces the overall mortality rate.

## Introduction

Acute type A aortic dissection (ATAAD) is a surgical emergency that can involve the superior mesenteric artery (SMA). Obstruction of the blood supply to the SMA could lead to malperfusion of the abdominal viscera, further causing intestinal ischemic necrosis, which results in serious consequences. Studies have demonstrated that mesenteric malperfusion is an independent predictor of death and is the most deleterious ischemic end-organ complication of ATAAD [1–4].

Acute type A aortic dissection complicated by mesenteric malperfusion has a poor clinical prognosis. Traditional emergency central aortic repair has a mortality rate ranging from 33-100% [1, 2, 5–10]. Surgeons are reluctant to perform open surgery in such critically ill patients because of the poor outcomes and formidable mortality of traditional surgical treatment. According to International Registry of Acute Aortic Dissection (IRAD) statistics, compared with patients without comorbid mesenteric malperfusion, patients who presented with mesenteric malperfusion were less likely to receive surgical treatment and more likely to receive only medical or endovascular therapy [4]. Although the mortality rate of medical treatment can be as high as 95.2-100% [4, 6], the mortality rate of endovascular treatment alone can be up to 72.7% [4].

As the above studies show, the treatment of ATAAD with mesenteric malperfusion is still difficult, and the results are not satisfactory. The optimal treatment strategy and the best intervention time for patients with ATAAD complicated by mesenteric malperfusion remain controversial. There are two critical but conflicting issues in the treatment of these patients, namely, aortic rupture and intestinal ischemic necrosis, both of which could lead to death. Many authors advocate for immediate central aortic repair, as these researchers hold the traditional view that the rapid restoration of true lumen blood flow can alleviate malperfusion in all distal aortic branches, but the clinical outcomes of immediate proximal aortic repair are suboptimal, and the mortality rate is extremely high [1, 2, 5–10].

In recent years, it has been gradually recognized that mesenteric malperfusion is a more important cause of eventual death in these patients. Moreover, the invention of endovascular techniques has provided an alternative minimally invasive approach for superior mesenteric artery revascularization, and surgeons have begun to try to apply interventional techniques instead of performing open surgery to address the superior mesenteric artery occlusion. Currently, a total of two strategies have been developed for the treatment of this disease. The first approach is to reperfuse the ischemic abdominal viscera first, followed by delayed surgery only after organ function is restored [6, 11, 12], and the other approach is to perform a simultaneous hybrid procedure [7, 13]. However, neither approach takes into account the relationship among the pattern of superior mesenteric artery anatomic obstruction, the duration of intestinal ischemia and the function of visceral organs.

Before 2021, emergency central open repair was routinely applied in Xiamen cardiovascular hospital for patients with ATAAD complicated by mesenteric malperfusion, with unsatisfactory clinical results. Thus, according to the pathophysiological mechanism by which irreversible intestinal injury occurs after 6 h of complete superior mesenteric artery occlusion [14, 15], we proposed a visceral function-focused 6-h time window strategy. Since 2021, we have performed emergency open surgical repair for all patients with an acute symptom onset within 6 h, and computed tomography angiography is performed immediately if malperfusion is suspected after surgery, and further interventions are initiated for residual lesions. For patients with a disease course beyond 6 h, we first restore superior mesenteric artery perfusion and initiate corresponding medical and surgical treatments for infectious shock and multiple organ failure. Only after visceral organ function has been restored do we proceed to open-heart surgery.

This study systematically reviewed and compared the clinical data of patients with ATAAD complicated by mesenteric malperfusion who received treatment at Xiamen cardiovascular hospital in two different periods. Our novel treatment strategy for mesenteric malperfusion is presented in detail, as well as the advantages and clinical outcomes of this strategy. We introduce our therapeutic strategy that focuses on the function of visceral organs in combination with the type of SMA occlusion and the pathophysiology of intestinal ischemia, hoping that this novel strategy could provide a better therapeutic benefit for this formidable disease.

## Materials and methods

### Study population and diagnostic criteria

1. Inclusion and exclusion criteria.

Patients admitted to Xiamen cardiovascular hospital for the treatment of ATAAD complicated by mesenteric malperfusion from 07.2018 to 06.2023 were enrolled. Patients who presented with coronary or cerebral malperfusion and patients with hemodynamic instability due to pericardial tamponade and acute cardiac dysfunction were excluded.

2. Definition and classification of ATAAD and mesenteric malperfusion.

Acute aortic dissection was defined as a new onset within 14 days [16]. Mesenteric malperfusion was defined as intestinal dysfunction or necrosis due to inadequate mesenteric blood flow, manifesting as corresponding physical signs and symptoms and with clinical evidence of inadequate blood flow.

Superior mesenteric artery occlusion due to ATAAD can be divided into three types according to the anatomic structure: the dynamic, static and mixed types. Static obstruction is caused by either an intimal flap obstructing the ostium of the artery or a false lumen protruding into the branch vessel with thrombosis, resulting in superior mesenteric artery occlusion. Dynamic obstruction is due to insufficient blood flow through the true lumen, and the pressure difference between the false lumen and the true lumen causes the false lumen to prolapse into the ostium of the SMA, causing obstruction. When the heart rate and blood pressure are controlled and blood flow through the true lumen is increased, the pressure in the true lumen exceeds that in the false lumen, the ostium of the SMA would reopen [17].

3. Clinical features and diagnostic criteria of mesenteric malperfusion.

The clinical presentation of mesenteric malperfusion is varied and atypical, and there is a lack of specific laboratory tests to accurately identify intestinal ischemia [18]. The common clinical manifestations include abdominal tenderness, intestinal paralysis, and infectious shock. Abnormal laboratory parameters include leukocytosis, lactic acidosis, abnormal liver and renal function tests, and elevated amylase levels. Among these manifestations, abdominal pain, although important, is a nonspecific symptom of acute mesenteric ischemia. In a large sample study conducted by the IRAD, 41.5% of patients with mesenteric malperfusion did not present with abdominal pain, while 24.2% of patients without mesenteric malperfusion presented with abdominal pain [4, 19]. Thus, the clinical diagnosis of mesenteric malperfusion relies mainly on the imaging evidence of superior mesenteric artery occlusion shown by contrast-enhanced computed tomography, and it also needs to be combined with typical clinical symptoms and laboratory findings for comprehensive judgment.

### Treatment protocol

**Fig. 1:**
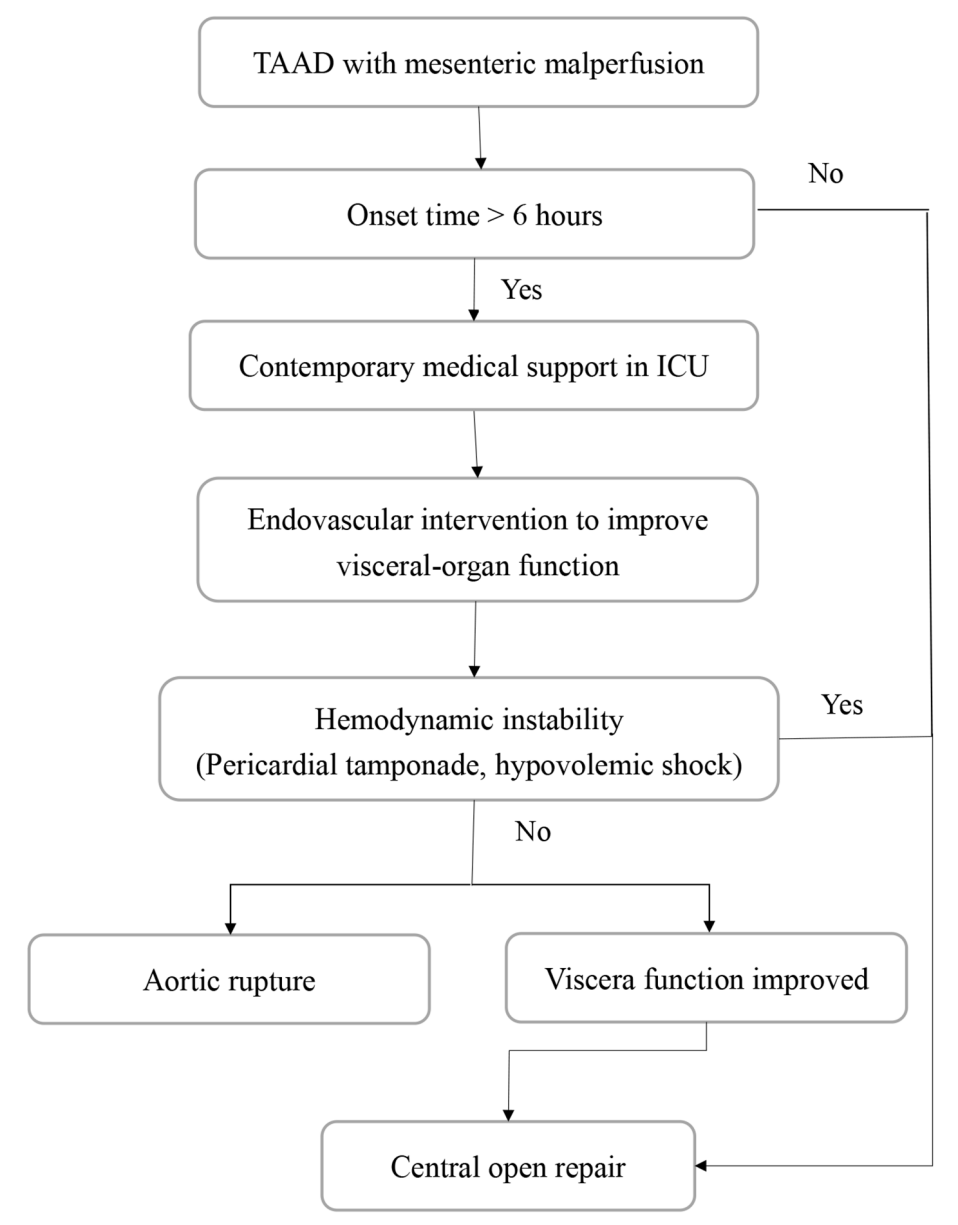
Flow chart of our strategy for mesenteric malperfusion. Additional, after central repair, immediate computed tomography angiography is performed in patients with symptom onset within 6 h if malperfusion is suspected after surgery

A flowchart was used to depict our diagnostic and treatment process (Fig. 1). In the phase I (07.2018–12.2020), emergency central aortic repair was performed for all enrolled patients. In phase II (01.2021–06.2023), we introduced a novel 6-h time window strategy that focused on the function of visceral organs. Studies have shown that irreversible intestinal injury occurs within 6 h of complete arterial occlusion [14, 15]. Therefore, we chose 6 h after acute onset as the time window for intervention based on the pathophysiology of mesenteric ischemia. Patients with symptom onset within 6 h underwent emergency central open surgical repair with an immediate postoperative imaging examination if malperfusion was suspected to remain to determine whether further interventions should be performed. Patients with an onset of more than 6 h were treated with endovascular surgery first to restore superior mesenteric artery perfusion. Simultaneously, treatment for infectious peritonitis, intestinal perforation, infectious shock, and multiple organ failure was provided, and open surgery was performed only after the patient’s visceral organ function had improved. The focus of treatment was on the restoration of organ function.

In clinical practice, when an endovascular intervention is performed for patients with an acute onset beyond 6 h, intestinal ischemia often develops into necrosis even with the successful recovery of superior mesenteric artery blood flow. Therefore, after successful endovascular therapy, it is necessary to assess and observe the condition of the visceral organs by a combination of physical examinations, laboratory indicators and CT imaging. Further approaches, such as exploratory laparotomy and the resection of necrotic bowels, must be performed when necessary.

The criteria for assessing the improvement of visceral organ function were a decreased blood lactate level, corrected metabolic acidosis, improved peritonitis and septic shock, and no clinical signs of persistent intestinal ischemia, which we considered to indicate corrected mesenteric malperfusion.

### Statistical analysis

IBM SPSS 24.0 software was used for data analysis, and *P* values of < 0.05 were considered to be significant. All quantitative data conforming to a normal distribution are expressed as the mean ± standard deviation, and data not conforming to a normal distribution are expressed as median values and interquartile ranges (IQRs). We used Student’s t test to compare continuous variables with normal distributions and the Mann‒Whitney U test to compare nonnormally distributed variables. The Fisher’s exact test was applied to analyze categorical data.

## Results

During the whole study period, a total of 734 patients who visited our hospital were diagnosed with ATAAD, of whom ten patients who refused surgery for various reasons (poor physical condition, advanced age), were not treated with our novel strategy, or did not undergo open surgery were excluded from the study. The proportion of patients with mesenteric malperfusion was 38/724 (5.25%).

The baseline characteristics and preoperative laboratory test results are shown in Table 1. No significant differences were found in the baseline data. In phase I (July 2018 to December 2020), all eleven patients with mesenteric malperfusion underwent emergency open surgery, three patients had an onset within less than 6 h, and only one patient died, while eight patients had an onset more than 6 h, and five died postoperatively. In phase II (January 2021 to June 2023), twenty-seven patients were diagnosed with mesenteric malperfusion, with sixteen patients with an onset greater than 6 h and eleven patients with an onset less than 6 h. In terms of the type of obstructions, a total of twenty-one patients had dynamic obstructions, eleven patients had static obstructions, and six patients had mixed obstructions. Fifteen, eight and four patients with an onset greater than 6 h had dynamic, static and mixed obstructions, respectively, and of the eleven patients with an onset less than 6 h, six had dynamic obstructions, three had static obstructions and two had mixed obstructions.

**Table 1:** Baseline data

Item	Phase I (11)	Phase II (27)	*P*
Onset time, n (%)			0.488
>6 h	8 (72.73)	16 (59.26)	
<6 h	3 (27.27)	11 (40.74)	
Male, n (%)	10 (90.91)	25 (92.59)	1
Age (years)	56 [20]	52 [22]	0.240
BMI (kg/m²)	25.30 ± 3.90	26.62 ± 3.82	0.345
Hypertension, n (%)	11 (100)	22 (81.48)	0.295
Diabetes, n (%)	1 (9.09)	4 (14.81)	1
Abdominal pain (n, %)	10 (90.91)	24 (88.89)	1
Bloody stool, n (%)	6 (54.55)	6 (81.48)	0.068
AST (IU)	289 [669]	99 [210]	0.288
ALT (IU)	153 [254]	98 [94]	0.246
TBIL (µmol/L)	25.35 ± 11.85	20.78 ± 11.05	0.265
Serum creatinine (mmol/L)	128.22 ± 50.70	138.45 ± 84.62	0.711
Max serum lactate preoperative (mmol/L)	3.95 ± 2.97	3.25 ± 2.21	0.432
LVEF (%)	60.91 ± 2.39	59.89 ± 4.34	0.469
Moderate to severe AR, n (%)	4 (36.36)	10 (47.62)	1
SMA obstruction types, n (%)			0.900
Dynamic	6 (54.55)	15 (55.56)	
Static	3 (27.27)	8 (29.63)	
Mixed	2 (18.18)	4 (14.81)	
Septic shock, n (%)	5 (45.45)	4 (14.81)	0.088
Renal insufficiency, n (%)	5 (45.45)	11 (40.74)	1
Hepatic insufficiency, n (%)	5 (45.45)	7 (25.93)	0.272
Paraplegia, n (%)	0	1 (3.70)	1
Low extremity ischemia, n (%)	3 (27.27)	4 (14.81)	0.390

Data presents as n (%), median [IQR], or mean ± SD. BMI: body mass index; AST: aspartate transaminase, ALT: alanine transaminase, TBIL: total bilirubin, LVEF: left ventricular ejection fraction, AR: aortic regurgitation, SMA: superior mesenteric artery, IQR: interquartile range, SD: standard deviation

Perioperative data for patients who underwent open surgery are shown in Table 2. All surgical patients underwent total arch replacement combined with elephant trunk implantation. In phase I, the surgical mortality rate was 6/11 (54.55%). All six patients with postoperative death had multiple organ failure triggered by intestinal necrosis, and all of them had bloody stools. In phase II, a patient with an onset within 6 h received immediate central open repair followed by postoperative CTA to evaluate the superior mesenteric artery flow. The mesenteric artery did not re-perfuse in this patient, as revealed on CTA, then stenting of the compromised branch was performed. Unfortunately, this patient eventually progresses to multiple organ failure and dead. Therefore, the surgical mortality rate was 1/23 (4.35%), showing a significant decrease compared to the first phase (*p* = 0.022), and the ICU stay was also shorter in phase II (13.82 ± 9.42 vs. 7.04 ± 4.62, *p* = 0.043).

**Table 2:** Perioperative data in the two phases (patients who underwent open repair only)

Item	Phase I (11)	Phase II (23)	*P*
Aortic arch strategy, n (%)			1
Hemiarch replacement	0	0	
Sun’s procedure*	11 (100)	23 (100)	
Aortic root procedure, n (%) Supracoronary anastomosis alone Aortic valve replacement Root reconstruction	6 (54.55) 4 (36.36) 1 (9.09)	14 (60.87) 4 (17.39) 5 (21.74)	0.471
Coronary artery bypass graft, n (%)	0	1 (4.35)	1
Cardiopulmonary bypass time (min)	190.73 ± 57.00	227.65 ± 92.26	0.233
Cross-clamping time (min)	121.55 ± 45.50	137.04 ± 44.37	0.352
Selective cerebral perfusion time (min)	15.45 ± 2.54	16.22 ± 4.33	0.593
Low cardiac output syndrome, n (%)	1 (9.09)	1 (4.35)	1
Reintubation, n (%)	3 (27.27)	1 (4.35)	0.089
ICU stay (days)	13.82 ± 9.42	7.04 ± 4.62	0.043
Surgical mortality, n (%)	6 (54.55)	1 (4.35)	0.022

*Sun’s procedure refers to total arch replacement combined with elephant trunk implantation

Data presents as n (%) or mean ± SD. ICU: intensive care unit, SD: standard deviation

The overall clinical outcomes for the two groups are shown in Table 3. The overall mortality rate was lower in phase II with the use of our novel strategy (54.55% vs. 18.52%, *p* = 0.047), and the proportion of patients with sepsis and multiorgan failure was also decreased, showing statistically significant differences (both 54.55% vs. 18.52%, *p* = 0.047).

**Table 3:** Overall outcomes in the two different phases

Item	Phase I (11)	Phase II (27)	*P*
Overall mortality, n (%)	6 (54.55)	5 (18.52)	0.047
Intestinal necrosis, n (%)	6 (54.55)	8 (29.63)	0.266
Cerebrovascular accident, n (%)	1 (9.09)	0	0.289
Paraplegia, n (%)	0	1 (3.70)	1
Pneumonia, n (%)	6 (54.55)	16 (59.26)	1
Renal insufficiency, n (%)	8 (72.73)	13 (48.15)	0.282
Requiring CRRT, n (%)	7 (63.64)	7 (25.93)	0.061
Sepsis, n (%)	6 (54.55)	5 (18.52)	0.047
Multiorgan failure, n (%)	6 (54.55)	5 (18.52)	0.047

Data presents as n (%). CRRT: continuous renal replacement therapy

The details of our novel strategy are shown in Table 4. None of the patients died due to aortic rupture, but 4 patients died due to organ failure during the waiting period. One patient with an onset of more than 6 h underwent laparotomy bowel resection. Four patients with an onset time of less than 6 h received endovascular treatment immediately after median sternotomy open aortic repair and survived. Among the patients with an onset time of more than 6 h, all underwent endovascular stent implantation and fenestration before open surgical repair. All patients with an onset time of more than six hours who had improved organ function and underwent open surgery survived.

**Table 4:** Reperfusion strategy in phase II.

Item	Number
Emergency surgery in patients with an onset < 6 h	11
Patients with an onset > 6 h during the waiting period	16
Aortic rupture	0
Organ failure	4
Underwent delayed surgery	12
Laparotomy bowel resection	1
Endovascular therapy after open repair in patients with an onset < 6 h	
Stent implantation	4
Fenestration	1
Reperfusion strategy for patients with an onset > 6 h	
Stent implantation	16
Fenestration	5

## Discussion

Aortic dissection is initiated when blood enters the medial layer of the aorta through a tear in the intima, which separates the aortic wall into false and true lumens under the impact of high velocity blood flow. During the downstream process of intima flap antegrade dissection along the aorta, the corresponding arterial branches are involved, which can cause obstruction of the branch arteries, resulting in malperfusion or even ischemic necrosis of the corresponding organs. Among them, mesenteric malperfusion is a rather rare but fatal condition. Studies of ATAAD complicated by mesenteric malperfusion are scarce due to its low incidence rate, and the vast majority of studies have been retrospective and conducted in a single center. These studies reported prevalences of only 1.4-14% [8, 11–13, 21–23]. In the study with the largest population, only 68 (3.7%) of 1809 patients had detectable mesenteric malperfusion [4]. In our study, mesenteric malperfusion accounted for 5.25% of all enrolled patients, the overall mortality rate (surgical mortality rate) in phase I was 54.55%, and the overall mortality rate in phase II was 18.52%. Therefore, despite its low incidence, ATAAD complicated by mesenteric malperfusion has a worse prognosis and high in-hospital mortality.

Currently, there is a large number of controversies in the treatment of this disease. Mesenteric malperfusion and aortic rupture can both have fatal consequences. The choice of treatment options, the restoration of mesenteric perfusion and avoidance of aortic rupture, which need to be considered comprehensively in the decision-making process, could be viewed as two sides of the same coin.

There are now four different treatment protocols for this disease. The first option is emergency open surgical repair. Conventional wisdom would suggest an emergency central open surgical repair in all patients with ATAAD (even in patients with mesenteric malperfusion) with the aim of preventing aortic rupture and treating mesenteric malperfusion. This strategy may be appropriate for relieving the dynamic obstruction of the SMA if mesenteric malperfusion has not progressed to the late stage involving intestinal necrosis. However, if end-organ injury is present or the SMA occlusion type is static, mesenteric malperfusion may not be able to be resolved by open surgical repair alone, so some surgeons perform SMA stent implantation if mesenteric ischemia and malperfusion persist postoperatively. However, this may still be too late if the patient’s intestinal function is already compromised at admission, as the real cause of death in such patients is often septic shock and organ failure due to intestinal necrosis. In addition, the prognosis of patients is seriously affected by the substantial trauma and ischemia‒reperfusion injury caused by open surgery, which also increases the difficulty in treating intestinal necrosis. Real-world studies have shown dismal results and high mortality rates using emergency central open repair, suggesting the need to consider alternative management strategies [1, 2, 5–10].

The second option is endovascular surgery. Fewer studies have reported on the outcomes of the endovascular repair of mesenteric malperfusion. As shown in a study with a large sample size by Eusanio et al., endovascular treatment alone (72.7%) had a better outcome than medical drug therapy (95.2%), but the mortality was still at an extremely high level [4].

The third option is hybrid surgery, which combines open surgical repair with endovascular procedures and has a lower mortality rate [7, 13]. However, hybrid operating rooms are expensive to establish and maintain, and they are not yet widely used. A very small number of studies could be retrieved because only a few medical centers have a hybrid operating room. Moreover, in patients with severe SMA involvement that has already progressed to the late stage of malperfusion and severe organ function impairment, the risk of death is relatively high. Because infectious shock and the systemic inflammatory response have not been corrected, surgery and ischemia‒reperfusion injury could further exacerbate trauma and adversely impact clinical outcomes. In addition, this strategy may not prevent “doomed” patients from undergoing procedures that they would not benefit from. These patients presenting with end-stage organ failure who do not receive prior treatment to improve organ function often experience multiorgan failure and death, regardless of whether open surgical repair is performed. In these cases, open surgical repair combined with endovascular therapy increases the risk of patient harm and greatly increases the consumption of medical resources without increasing therapeutic efficacy.

The last strategy is performing endovascular reperfusion therapy first, followed by delayed open surgical repair. The main advocates of this strategy are the team at the University of Michigan, who recommend restoring visceral organ function before performing open surgery. This strategy has already shown a more favorable clinical outcome. However, all patients with ATAAD complicated by mesenteric malperfusion enrolled at their institution receive endovascular therapy first to restore organ perfusion. Thus, some patients with early-stage mesenteric malperfusion caused by dynamic obstruction of the involved superior mesenteric artery that has not yet developed into intestinal necrosis, which could be corrected by emergency open surgical repair, are also treated with endovascular therapy. During this period, there is also a potential risk of aortic rupture. 33% of the patients in Patel’s study died during the waiting period, and 38% of the patients in Yang’s study died preoperatively [6, 11, 12].

To treat ATAAD complicated by mesenteric malperfusion, the importance of intestinal ischemia treatment is now at the forefront, and many scholars believe that only after the correction of mesenteric malperfusion can a favorable surgical outcome be achieved [7, 12, 13, 24]. However, neither hybrid surgery nor endovascular therapy followed by a delayed open repair strategy takes into account the relationship between visceral organ function and ischemic time or the differences in the treatment of dynamic and static obstructions. When end-stage intestinal ischemia with clinically evident intestinal necrosis occurs, an endovascular approach to reperfuse the ischemic organ as an initial procedure may be more likely to achieve patient survival and prevent ineffective open aortic repair in these high-risk individuals. Instead, in patients with a short duration of ischemia, emergency open surgical repair should be performed first in those with malperfusion but without significant end-organ dysfunction, which can save the vast majority of patients with dynamic obstruction.

Previous studies have already shown that irreversible intestinal injury occurs beyond 6 h of complete SMA occlusion [14, 15]. Therefore, we proposed a visceral function-focused strategy with a 6-h time window based on the relationship between the duration of intestinal ischemia and visceral injury. In patients with an onset beyond 6 h, prolonged severe intestinal ischemia often leads to irreversible intestinal necrosis with toxic intestinal paralysis, hyperlactatemia, and infectious shock. In the treatment of such patients, it is necessary to address mesenteric malperfusion first and to restore the function of the visceral organs. Measures such as SMA stent implantation, exploratory laparotomy, and resection of necrotic bowel are recommended. Delayed open surgical repair can be performed only after the restoration of organ function. For patients with a short duration of ischemia, direct open surgical repair can save the majority, as dynamic obstruction is the main cause of malperfusion, accounting for approximately 80% of all cases [25]. Computed tomography imaging is reviewed promptly when malperfusion is suspected postoperatively, and, if necessary, endovascular therapy is performed for static obstruction that is not corrected by open surgery. When SMA occlusion causes intestinal ischemia with an onset of more than 6 h, we apply the delayed surgical repair strategy that restores perfusion to the affected organ first and waits for the improvement of intestinal function before open surgical repair. In phase II, we applied this approach and achieved excellent clinical outcomes. Our first-stage operative mortality rate was 54.55%, and the second-stage operative mortality rate was 4.35%, from which we could see a significant decrease. In patients with an onset of more than 6 h, no patients died from aortic rupture, and only 4 patients died due to organ failure during the waiting period in phase II. The surgical success rate in patients survive the waiting period was 100%, from which we could see a significant increase in the operative success rate. These outcomes were also at a better level when compared with the study of Yang et al. [6, 11, 12].

Surgeons may be concerned about the risk of aortic rupture during the waiting period after restoring mesenteric perfusion and improving intestinal function. Previous studies have suggested that the risk of aortic rupture is high and that half of patients die from aortic rupture within 48 h [26]. Death occurs in 1–2% of patients per hour in the first 48 h after symptom onset [27, 28]. This view has been held for nearly half a century. However, recently, Yang B and other research teams mentioned the finding that the probability of rupture during the waiting period was lower than the mortality rate of end-organ failure and even more clearly indicated that the probability of aortic rupture showed a significant decrease over time [11, 20, 29]. In our practice, our results demonstrate that the probability of in-hospital aortic rupture under contemporary intensive medical treatment has been greatly reduced. No patients developed aortic rupture during the waiting period in this study. The reasons for this may be related to more aggressive medical treatment, as well as better intensive care. Together, intravenous sedation and analgesia as well as tracheal intubation and bedside hemodialysis filtration therapy contribute to a decrease in the probability of rupture during the waiting period. Therefore, we insist that the application of our novel strategy is safe and that the risk of aortic rupture is minimal.

There are still some limitations of our study. This was a single-center retrospective study; coupled with the low prevalence of mesenteric malperfusion, this results in an extremely limited study population. Because patients with ATAAD are in critical condition, it is not possible to perform a randomized controlled trial. Despite the shortcomings, we are the first to apply a strategy in which the intestinal ischemia duration, the type of superior mesenteric artery occlusion and visceral organ function are comprehensively considered in the treatment of ATAAD complicated by mesenteric malperfusion. The clinical results of our strategy are favorable, and we believe that our strategy can indeed improve the prognosis of patients with ATAAD complicated by mesenteric malperfusion. This strategy allows us to perform timely open surgical repair to resolve mesenteric malperfusion (dynamic obstruction) in most cases before malperfusion has progressed to intestinal necrosis and to avoid ineffective open surgical repair in patients with irreversibly compromised mesenteric function. We expect that this approach can be validated by studies involving more centers in the future.

## Conclusions

Our tailored strategy for patients with ATAAD complicated by mesenteric malperfusion can achieve a better clinical outcome. Not only does this strategy improve the surgical success rate, but it also reduces the overall mortality rate.

## Data Availability

Data sharing is not applicable to this article, as no datasets were generated or analyzed during the current study.

## Abbreviations

ATAAD: Acute type A aortic dissection
SMA: Superior mesenteric artery
IRAD: International Registry of Acute Aortic Dissection
